# Open Science, Health Data and Epistemic Harms: A Multidisciplinary Reflection

**DOI:** 10.5334/dsj-2026-015

**Published:** 2026-04-10

**Authors:** Tatenda Chatikobo, Frances Griffiths, Nikita Hayden, Gary Leeming, Ankita Mishra, Eva Morris, Luca Schirru, Nathanael Sheehan, Andrew Williams, Sharifah Sekalala

**Affiliations:** University of Warwick, United Kingdom; University of Sheffield, United Kingdom; University of Liverpool, United Kingdom; University of Sheffield, United Kingdom; University of Oxford, United Kingdom; Brazilian Copyright Institute, Brazil; Technical University of Munich, Germany; University of Warwick, United Kingdom

**Keywords:** Open Science, epistemic harms, social justice, health data, research data, public health

## Abstract

Open Science (OS) promises to democratise knowledge and reduce epistemic inequalities. However, a critical analysis reveals the potential of OS to amplify structural vulnerabilities, especially for people and communities already at the margins. With a particular focus on health data, this interdisciplinary essay examines how OS infrastructures perpetuate epistemic harms through the dominance of Eurocentric knowledge norms, legal regimes and corporate capture. Amidst the rapidly evolving health and data landscape, realising the social justice potential of OS, especially in healthcare, demands moving beyond techno-optimism to approaches that centre plural epistemologies, relational accountability and community empowerment.

## Introduction

Article 27 of the 1948 Universal Declaration of Human Rights recognises everyone’s right to access and the benefits of science. Yet closed models of science have hampered this ideal (Azoulay, 2020), leading to inequalities between countries and to weakened coordinated responses to crises, including climate change, biodiversity degradation and pandemics. Intergovernmental institutions such as the United Nations Educational, Scientific and Cultural Organization (UNESCO), the World Health Organization (WHO), the European Organization for Nuclear Research (CERN), and the Office of the United Nations High Commissioner for Human Rights (OHCHR) have called for the adoption of open science approaches to promote universal access to scientific progress. Open Science (OS) is often defined as a set of practices and a movement for making scientific knowledge openly available, accessible and reusable for the collective benefit of society (UNESCO, 2022). OS practices include granting free access to: scientific data through Open Data, peer-reviewed research literature through Open Access and programming code through Open-Source Software. They also involve participatory research methods through Citizen Science (Cole *et al*., 2024; COS, n.d.). At the supranational level, UNESCO established a global framework on OS in 2021, serving as a roadmap for countries and regions to develop an enabling policy environment, support infrastructure investments, and promote partnerships for OS (UNESCO, 2021).

Within health, the concept of openness to diverse knowledge systems, technologies and data has the potential to lead to cost efficiencies as scientific discoveries become more easily shareable, verifiable and reproducible (Hetu, Koutouki and Joly, 2019; Wyber *et al*., 2015; D’Agostino *et al*., 2018). However, achieving this ideal requires a critical awareness of the ways openness can also amplify harms. OS policy across actors—from states, intergovernmental organisations and research institutions—often reflects an inherently optimistic assumption that openness is unproblematically beneficial (Chtena *et al*., 2025). Emerging evidence highlights how unrepresentative datasets, extractive models of research data collection and commercial actors’ infrastructural capture can undermine equity, participation and public interest in OS (Hofmann, 2022; Ross-Hellauer *et al*., 2022). For instance, there are also concerns that Open Access (OA) through Article Processing Charges has become an attractive business model for commercial publishers, while sustaining inequalities between researchers from high-income countries (HICs) and low- and middle-income countries (LMICs) (Hagner, 2018).

In terms of health, a critical analysis of OS has exposed unique and complex issues. With recurring tropes about data as the *new oil* and *new gold*, there is an increasing value placed on health data, not only for its potential to inform health interventions but also to create economic opportunities (see Stacey and Milmo, 2025). The economic logic of health data, often reflected in research and development policy discourse, raises concerns that such approaches may ultimately empower data corporations without substantial benefits to patients and communities (Byczkowski, 2025; Pal, 2025). There are also emerging concerns that health data may also be used in ways that exacerbate existing vulnerabilities. For example, predictive health algorithms trained on biased datasets may produce inaccurate risk assessments for minority populations, leading to downstream disparities in healthcare (Cross, Choma and Onofrey, 2024).

This essay offers a critical reflection on the current moment and trajectory of the OS approach and movement. We achieve this by making two significant contributions to current scholarship. First, drawing on our diverse fields of practice, including law, epidemiology, public health, psychology, data science, disability studies and data justice, as well as existing literature, we provide a critical reflection on the complex and often contested processes of OS. With a particular focus on health data and participatory research experiences, our critique examines issues of epistemic harms, which are restrictions on access and benefits to knowledge exchange (Hardwick, 2019), reproduced by current OS approaches. Second, we provide an imaginative vision for the future of OS as a field and the implications for foundational commitment to epistemic justice in a rapidly changing world.

The essay draws on multidisciplinary perspectives on OS explored during a global and public health workshop on *Open Science Architectures: Lessons for Global Health Research and Innovation*, hosted by the Centre for Global Health Law at the University of Warwick, UK, in September 2025 (see Appendix A). The workshop featured two panels: one critically examining key trends in OS and how they are transforming the health research and innovation landscape, and another on the implications of technological developments such as Artificial Intelligence for creating inclusive, accountable and ethical OS infrastructures for health justice. The workshop also provided an opportunity for us to consider and discuss ways to strengthen OS movements to promote health justice across dimensions such as gender, race, economic status, (dis)ability and geography. As a result of the discussions, all participants agreed to co-author a publication to share their reflections.

## Context: The Promise and the Paradox

The principal benefits of OS can be summed up this way: first, OS accelerates the development of solutions by making research data, code, and protocols widely available for reuse (Wehn *et al*., 2024). Within the context of health, OS has enhanced preparedness, rapid responses and localised adaptations of interventions through open epidemiological data and biomedical resources (Wells *et al*., 2016). For instance, the International Nucleotide Sequence Database Collaboration (INSDC), composed of the European Nucleotide Archive, the National Center for Biotechnology Information, and the DNA Data Bank of Japan is the largest open-access repository of nucleotide sequences and biological annotations. During the COVID-19 pandemic, INSDC provided multiple real-time access platforms for genome sequence information, facilitating critical work for public health research such as variant tracking and vaccine development (Sheehan, Botta and Leonelli, 2024; Sayers *et al*., 2025). Second, by making research open and shareable, OS practices can enhance accountability, foster public and societal trust, and broaden participation beyond traditional scientific communities to also include patient and participant communities, civil society organisations, and activists (UNESCO, 2022). For example, the Civic Data Cooperative in Liverpool, UK, is creating a digital commons as a shared resource to guarantee that data from residents of the Liverpool City Region benefits their communities through cooperative decision-making and transparent governance (Civic Data Cooperative, n.d.). Third, by removing barriers to access and participation, OS can also serve as an ethical model for redress, closing the gaps between science and society by aligning scientific research priorities with societal needs (Grand *et al*., 2012).

Despite these benefits, OS is increasingly operating within a political economy of interests that are contrary to the aspirations of openness and equity (Bezuidenhout *et al*., 2017a; Leonelli, 2022; Bezuidenhout, 2025). OS has the potential to reproduce hierarchical and dependency relationships (Serwadda *et al*., 2018). This is due to the uneven distribution of technological resources, financing mechanisms and human resources across contexts, which remain skewed towards high-income areas (Bezuidenhout *et al*., 2017b). From our experiences, this divide, commonly characterised as geographical Global North and South, is not only vertical (in the sense of HICs and LMICs) but also horizontal. Race, gender, (dis)ability, and socio-economic status often shape differential benefits, even for communities within the same geographical context. For example, concerns have been raised that the lack of diversity in genomic datasets can reinforce existing class and racial inequalities by developing biomedical treatments that benefit certain groups over others or by producing inaccurate predictions about risk factors (Omeranovic *et al*., 2025).

Without enforceable reciprocity mechanisms, open datasets derived from marginalised communities often end up in commercial value chains and intermediaries, yielding limited to no tangible returns for the communities themselves (Madianou, 2019). Wealthy corporations and other private actors frequently benefit from intellectual property (IP) by gaining greater control over research data, leading to enclosures and thereby undermining the fundamental principles of OS (De Beer and De Koker, 2024; Knoche and Fuchs, 2020). OS has been co-opted into platform capitalism, serving as an infrastructure for data colonialism (Dutta *et al*., 2021; Mirowski, 2023). For instance, open repositories, publishing platforms and data sharing agreements are often governed by corporate or elite institutions that continue to privilege certain epistemic norms of objectivity, quantification and decontextualisation that emerge from Northern, reductionist and individualistic knowledge traditions (Leonelli, 2023).

Apart from the outcome of OS having the potential to structure inequalities, there is also a broader issue of how the process itself shapes access to research data. Through the bureaucratisation of research data infrastructures, OS risks being subsumed under managerial rationalities that prioritise compliance and auditability over attending to existing harms (Pievatolo, 2020). In this process, both access to research data and opportunities for participation are mediated by exclusionary institutional norms and adherence to standardised protocols, rather than genuine, collaborative exchanges of benefits (Leonelli, 2022). Despite claims of democratised access and transparency, OS infrastructures remain deeply embedded in technical and complex design logics. This highly specialised nature of research data and systems can create barriers to engagement, effectively excluding non-technical interest groups from participating meaningfully in oversight and accountability processes, as well as in shaping OS frameworks (Sheehan and Leonelli, 2025). Without challenging inequalities that prevent meaningful participation and sustain knowledge hierarchies in OS, the promise of openness can therefore become performative and data and data uses can reinforce exploitative relations in a social setting (Goldensher, 2023).

## Reflections: OS and Epistemic Harms

But what have been our experiences of the tensions between the social justice potential of OS and the risk of epistemic harms? First, relational dynamics play a significant role in shaping OS outcomes. The sharing of epidemiological data between researchers and clinicians has the potential to transform diagnosis and treatment. For example, initiatives such as the Get Data Out programme and the Health Data Research Hub for Cancer in the UK facilitate access to high-quality cancer datasets for researchers. In our experience, effective data sharing between researchers and clinicians requires strong, mutual trust that access to datasets is accompanied by responsible and ethical use for the ultimate benefit of patients. This trust is increasingly under threat due to the rising influence of commercial tech infrastructure mediaries in the handling and processing of health data. We are concerned that such private actors may capitalise on openness to advance market priorities that do not align with public benefit goals.

Second, the question that continually arises from our collaborative and participatory approach to research is not simply how open, but open for whom and on whose terms (see also Dominik *et al*., 2022)? As several of our partners in participatory research projects have reminded us, openness can reproduce inequities in new technologically mediated ways. In one of our research programmes, our community partner based in the geopolitical South has cautioned us that *openness* can feel like extraction to them when local knowledges are digitised without community governance and participatory stewardship. This is especially relevant in the context of intersectional health data. For example, non-university/community partners across one of our research projects have also warned that data on disability, once decontextualised, can be reinterpreted through ableist frameworks and colonial gazes that construct disability—particularly in Southern contexts—through a deficit lens, erasing the local meanings and lived realities. In such cases, withholding data can be an act of care grounded in trust, dignity and accountability.

Third, OS has been built mainly on quantitative paradigms, creating profound challenges and risks for qualitative and co-produced research. While quantitative data can be effectively anonymised through depersonalisation, qualitative data are relational, contextual and deeply personal. Our experiences archiving in-depth qualitative data have revealed insurmountable challenges related to protecting participants’ anonymity, respecting their stories, providing sufficient context for the data to be useful and ensuring tangible benefits for community members specifically. For researchers, preparing qualitative data for proper archiving to address the challenges above requires significant time investment that is rarely accounted for in research budgets. There are also significant ethical issues surrounding informed consent—even when participants understand how their data will be archived, they cannot predict how it will be used, or potentially misused, by other researchers. Without reimagining OS through what we call ‘humanising’^1^ or ‘cripping’^2^ OA and developing truly inclusive and relational infrastructures with marginalised communities, the archiving of sensitive data may become a new frontier for deepening, rather than dismantling, inequalities.

Fourth, we view legal frameworks, especially intellectual property rights (IPR), as both facilitating and constraining OS’s transformative ambitions. While the relationship between stronger IP rights, innovation, and economic development remains disputed (Chang, 2003; Dosi and Stiglitz, 2014), requiring developing countries to adopt expansive IP regimes may reinforce epistemic inequalities, particularly in relation to OS. This reflects both the limited suitability of such systems in technologically developing contexts and the broader constraints associated with institutional transplants (Dosi and Stiglitz, 2014). One-size-fits-all approaches often fail to account for local priorities and dynamics in OS (Schirru and Albagli, 2026). Furthermore, we note that even when transformative approaches to data governance are envisioned to promote OS, the role of civil society and grassroots movements in shaping more equitable knowledge ecologies is often overshadowed by an overemphasis on researchers. Yet, civil society organisations and community networks can broaden the scope of OS by supporting more participatory models of knowledge creation and dissemination, particularly in contexts where research infrastructures are unevenly distributed.

## Conclusions: OS Future Trajectories

As OS becomes an integral approach shaping contemporary research practice, we offer some reflections to guide the development of this emerging field.

Applying lessons from Critical Data Studies and Science and Technology Studies, a more equitable OS approach needs to centre the conceptual definition of data, not as neutral and objective, but rather as relational and situated. This acknowledges that data is always generated from specific locations, embedded perspectives and power relations. By doing so, OS can be more reflexive of the embedded yet subtle power dynamics in research data, acknowledging how they emerge from discourses, institutions and architectural forms that serve specific power structures. This requires confronting the problematic values underpinning the epistemic foundations of OS itself. Such an approach can reveal the ways openness can, in some contexts, lead to erasure or a colonial gaze.

Integrating participatory research methodologies guided by principles of collective benefit, authority to control, responsibility and ethics (CARE) offers a pathway towards more ethical data practices by centring communities in OS (see Carroll *et al*., 2020). For instance, Kukutai and Taylor (2016) proposed a data sovereignty approach that empowers Indigenous communities to make exclusive determinations over how data about their communities, lived experiences, and territories are managed in line with their inherent rights, traditions and responsibilities. As a generative space of learning, OS must also draw from Southern traditions of openness grounded in reciprocity, relational accountability and collective benefit. After all, the idea of openness is not new or limited to the dominant Northern conception; Indigenous knowledge productions have long traditions of openness and collaboration (Dutta *et al*., 2021).

A key lesson for us is that *availability of data is not the same as accessibility*, as that would require formats that meet diverse needs in terms of textual, visual, auditory and language formats. Therefore, accessibility cannot be an afterthought added to OS agendas; it must be an organising principle through which openness is enacted as collective care. Work should also be done on the potential democratising impact of OS where it can be more easily refused if it is not aligned to local values and needs. This is while recognising that currently, there is a significant imbalance in access, skills and benefits related to science and technology innovations.

We need to consider a more robust OS accountability infrastructure. Given that some of the potential harms of OS are symptomatic of broader systemic inequalities, a more deliberate accountability mechanism is necessary to identify and address these harms as a constitutive element of OS itself. Such an infrastructure could include stakeholders who have been influential and effective in driving social change in different domains, such as local journalists, rights advocacy groups, activists and labour unions. Currently, the democratic spaces in which these stakeholders work are increasingly under threat globally, with relentless attacks on press freedom and grassroots social justice movements, which might limit opportunities for solidarity. In particular, the erosion of media independence and pluralism undermines the watchdog function essential for holding researchers and institutions accountable to the public interest.

There is a need to envision a new funding model for OS. While there is no consensus yet on what an alternative funding model for OS could look like, it is clear that the current model is not working as it is heavily dependent on external support rather than being self-sustaining. Here, there are opportunities to learn from models that have worked in other contexts within OS and more broadly in creating social benefit ecosystems. Sustaining OS in a just and inclusive way therefore means investing in locally governed infrastructures, such as repositories managed by communities, ethical review processes that foreground epistemic justice, and capacity-building initiatives that redistribute resources towards marginalised groups.

## Footnotes

1 Humanising refers to ‘principles and practices of empathy, dignity, compassion, kindness and recognition’ (Goodley, 2025).

2 Cripping Open access involves critically interrogating and redesigning open access infrastructures, policies, and practices to move beyond compliance-based accessibility towards transformative inclusion. Drawing from crip theory and disability justice (Kafer, 2013; Mingus, 2011; Piepzna-Samarasinha, 2018), it recognises that knowledge production and access are shaped by ableist norms that privilege speed, standardisation, independence and text-centric communication. Cripping OA therefore prioritises flexible formats, multimodal access, relational participation and the legitimacy of non-normative knowledge practices, while foregrounding care, interdependence and the right to refuse participation or disclosure.

Additional File The additional file for this article can be found as follows:

- **Appendix A.** Workshop Programme & Panel Scope. https://doi.org/10.5334/dsj-2026-015.s1
